# Laser Vestibuloplasty for Peri-implant Gingiva Implementation in the Atrophic Mandible of a Medically Compromised Patient

**DOI:** 10.7759/cureus.7349

**Published:** 2020-03-21

**Authors:** Domenico De Falco, Daniela Di Venere, Gianfranco Favia

**Affiliations:** 1 Dentistry, University of Bari Aldo Moro, Bari, ITA

**Keywords:** vestibuloplasty, perimplant gingiva, diode laser, oral surgery

## Abstract

Despite the good clinical outcomes of the conventional technique of vestibulopasty performed with a scalpel, patients frequently perceive pain and discomfort after surgery. Diode laser use in oral surgery is, instead, well tolerated as it is mini-invasive and with a very low occurrence of post-surgical complications. In addition, the lack of bleeding during surgery and the reduced/absent post-surgical edema makes diode laser surgery the most suitable for medically compromised patients. We report a case of vestibuloplasty performed by diode laser in a patient on therapy with warfarin and without drug discontinuation.

## Introduction

It is generally accepted that the long-lasting duration of dental implants is strictly related to the quality and quantity of the keratinized gingiva [1]. Prosthetic rehabilitation of atrophic mandible by implant-retained overdenture is frequently associated with a reduced or poor quality adherent gingiva, thus needing its implementation. Among all techniques reported in the literature, the conventional technique of vestibuloplasty performed with a scalpel is surely the most used, although characterized by pain and discomfort perceived by patients. Above all, it is related to post-surgical edema and difficulties in swallowing, chewing, and speaking [2-6]. Such complications could become more evident in medically compromised patients (e.g. on anticoagulant therapy) [7]. The use of a laser with good surgical capabilities (especially contextual cut and coagulation) may surely improve clinical outcomes in patients needing vestibuloplasty [5,6,8]. We report the case of a patient who underwent vestibuloplasty by diode laser to implement the keratinized gingiva around dental implants in an atrophic mandible; the patient was in therapy with warfarin and without drug discontinuation.

## Case presentation

**Figure 1:**
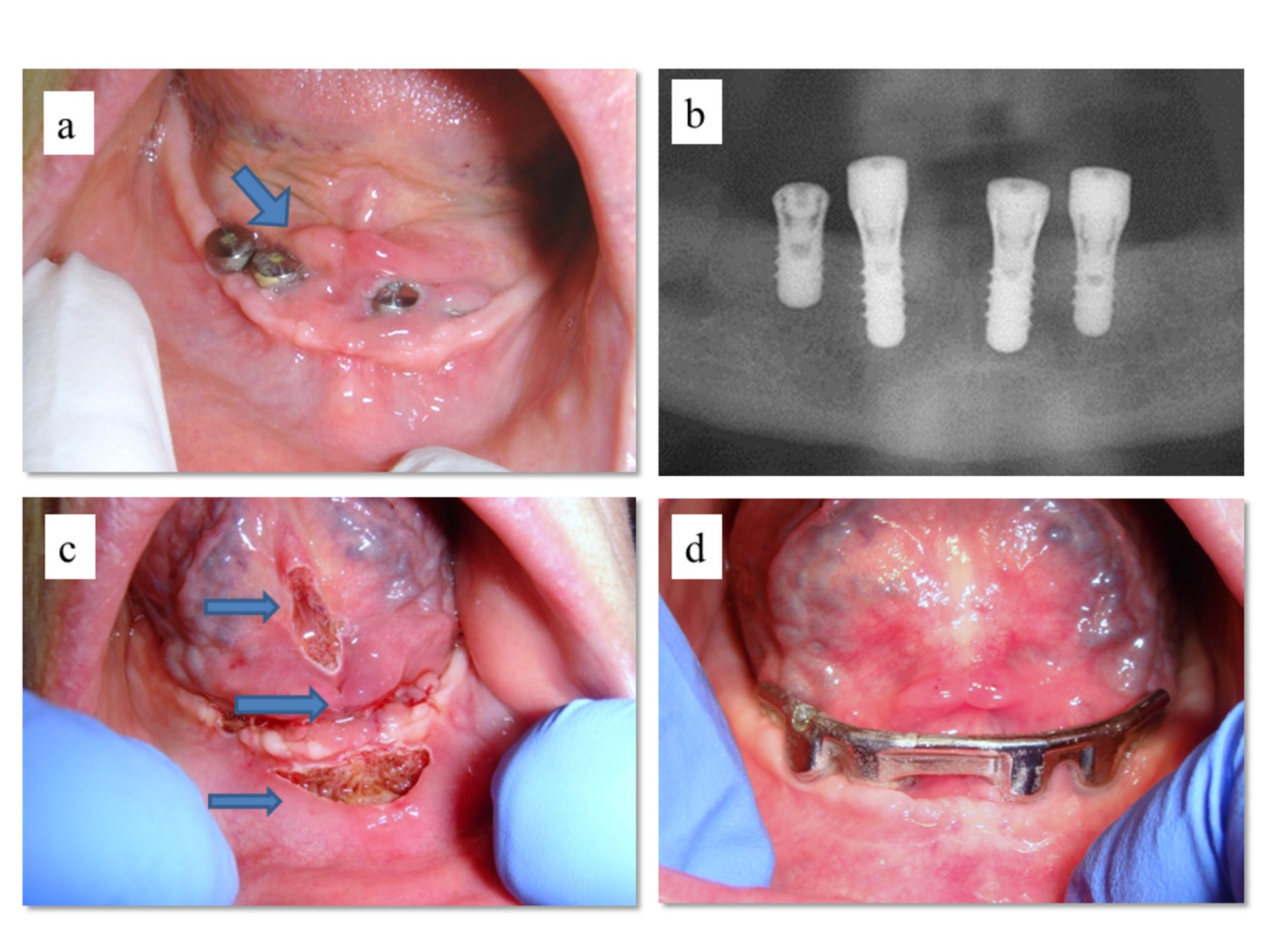
Clinical appearance of soft tissues around the dental implants in the atrophic mandible of the patient in anticoagulant therapy three months after insertion (a,b); caruncula sublingualis (arrow) appeared in proximity of the gingiva, resulting un-adherent and mobile around fixtures; diode laser surgery of both lingual and buccal vestibula along with tongue frenulotomy (arrows) (c); clinical appearance after 15 days showing implementation of the peri-implant keratinized gingiva, with creation of a sufficient prosthetic space for the following rehabilitation (d).

The patient was a 75-year-old female who received four implants in the mandible for prosthetic rehabilitation by overdenture. She was in therapy with warfarin for chronic atrial fibrillation. Three months after the implant insertion, a clinical examination revealed the poor quality and quantity of keratinized gingiva around the fixtures. More precisely, it revealed the proximity of caruncula sublingualis and the absence of an inadequate deepening of both buccal and lingual vestibula of the mandible limited the prosthetic space and causing instability of the peri-implant gingiva (Figures 1a-1b). The patient was treated by diode laser surgery (wavelength 980 ± 10 nm; continuous wave, output energy 1.5 W) without warfarin discontinuation; healing caps were removed and the implant was re-covered with gingiva using stitches, and vestibuloplasty was performed (by using a diode laser) on both aspects of the mandible with contextual lingual frenulotomy (Figure 1c). No bleeding was observed during surgery and stitches were unnecessary; no post-surgical complications occurred. After 15 days, a complete resolution of all wounds was observable with an evident increase of the available prosthetic space (Figure 1d).

## Discussion

Anticoagulant therapies are usually modified/suspended in patients needing oral surgery procedures to prevent both intra- and post-operative bleeding [7,9]. The generally accepted advantages of diode laser use in oral surgery are the decreased need of anaesthesia, the lack of intra-operatory bleeding, the reduction of post-operative oedema, unnecessary stitches, and the acceleration of mucosa healing [8]. For such reasons, laser therapy represents a safe and predictable procedure for several surgical and not-surgical treatments in the oral cavity, e.g. periodontal decontamination, non-surgical drug-related gingival overgrowth treatment, surgical removal of benign proliferating lesions as well as of oral mucosa malignancies [10-14]. The use of diode laser for vestibuloplasty, in patients on anticoagulant therapy, is associated with a shorter operating time and fewer postoperative complications compared to conventional scalpel surgery including the unnecessary drug discontinuation [5,6].

## Conclusions

Among all lasers with proven surgical capabilities, the diode laser is widely used for the surgical excision of proliferating lesions and photocoagulation of small and large venous malformations in the oral cavity. Although vestibuloplasty may represent an invasive procedure in medically compromised patients, especially during possible intra-operative bleeding and the following post-surgical edema of the floor of the mouth, diode laser use allows to simplify such procedure in terms of operating time, invasiveness, really reducing pain and discomfort, while at the same time achieving excellent clinical outcomes.
